# Fundamental principles of an anti-VEGF treatment regimen: optimal application of intravitreal anti–vascular endothelial growth factor therapy of macular diseases

**DOI:** 10.1007/s00417-017-3647-4

**Published:** 2017-05-19

**Authors:** Paolo Lanzetta, Anat Loewenstein, The Vision Academy Steering Committee

**Affiliations:** 1 Department of Medicine – Ophthalmology, University of Udine, Piazzale S. Maria della Misericordia, 33100 Udine, Italy; 2 Tel Aviv Sourasky Medical Center, Tel Aviv, Israel

**Keywords:** Anti–vascular endothelial growth factor, Retinal disease, Treatment regimens, Visual acuity, Aflibercept, Treat-and-extend

## Abstract

**Background:**

Intravitreal anti–vascular endothelial growth factor (VEGF) therapy is now considered the gold standard for the treatment of various retinal disorders. As therapy has evolved, so too have the treatment regimens employed by physicians in clinical practice; however, visual outcomes observed in the real world have typically not reflected those reported in clinical trials. Possible reasons for this include a lack of consensus on treatment regimens and a lack of clarity about what the aims of treatment should be.

**Methods:**

The Vision Academy Steering Committee met to discuss the principles of an ideal treatment regimen, using evidence from the literature to substantiate each point. Literature searches were performed using the MEDLINE/PubMed database (cut-off date: March 2016) and restricted to English-language publications. Studies with fewer than ten patients were excluded from this review.

**Results:**

The Steering Committee identified the following four key principles for the ideal treatment regimen for anti-VEGF management of retinal diseases:

1. Maximize and maintain visual acuity (VA) benefits for all patients
2. Decide when to treat next, rather than whether to treat now
3. Titrate the treatment intervals to match patients’ needs
4. Treat at each monitoring visit.

**Conclusions:**

It is proposed that the adoption of a proactive and more personalized approach in the clinic such as a treat-and-extend regimen will lead to benefits for both the patient and the physician, through a reduction in the associated treatment burden and better utilization of clinic resources. Implementation of the four principles should also lead to better VA outcomes for each patient, with a minimized risk of vision loss.

## Introduction

The Vision Academy is a global group of ophthalmic experts whose goal is to identify and address unmet needs in ophthalmology. The Vision Academy Steering Committee (see Appendix) met in 2014 to discuss the principles of an ideal treatment regimen. This review documents the key points from that discussion, and provides evidence from the literature to support these recommendations. These principles were not conceived as a specific set of guidelines or an in-depth review of treatment regimens (which have been covered elsewhere [1]), but rather as a framework to encourage discussions between retinal specialists on how to achieve optimal outcomes for each patient while optimizing treatment and clinical practices.

### Anti-VEGF treatment of retinal diseases

Retinal disorders, including neovascular age-related macular degeneration (nAMD), macular edema following branch and central retinal vein occlusion (BRVO and CRVO), myopic choroidal neovascularization (mCNV) and diabetic macular edema (DME), are major causes of preventable vision loss and impairment worldwide [2–4].

AMD is the leading cause of blindness in the elderly [2] and affects 10–13% of adults aged 65 years or over in North America [5]. It is predicted that by 2020, approximately 196 million people will be living with this disease, as a consequence of an aging population [6]. In 2014, the worldwide prevalence of DME was estimated to be around 21 million [4], with this figure expected to rise to 100 million by 2030 [7], due to an increase in the incidence of diabetes. This in turn is due to a number of factors, particularly an aging and increasingly obese population. As the onset of diabetes and subsequent complications is most common in young or middle-aged groups, the disease affects patients during their productive working lives [4]. Visual impairment on a global scale thus translates into a significant economic burden, including direct costs of treatment, other direct costs associated with vision impairment (e.g., nursing home costs and guide dogs), and billions of dollars in lost productivity each year due to a reduced labor force [8].

Some of the early treatment options for these conditions, such as laser photocoagulation and photodynamic therapy (PDT), have since been associated with poor visual outcomes and risk of adverse effects [9–17]. While these may still have a limited role in the management of retinal disorders, the advent of intravitreal therapy specifically targeting VEGF-A has revolutionized the treatment of such disorders, providing rapid and sustained improvements in VA alongside favorable side-effect profiles.

Pegaptanib sodium (Macugen^®^), the first anti-VEGF agent to be approved for the treatment of nAMD [13], was designed to specifically target the VEGF-A 165 isoform [18–20]. However, experience has shown pegaptanib sodium to be less effective at improving visual outcomes than newer anti-VEGF agents that target all VEGF-A isoforms [21].

Bevacizumab (Avastin^®^) and ranibizumab (Lucentis^®^) are anti-VEGF agents that are used more frequently than pegaptanib. Bevacizumab is licensed for use in several adult cancers [13], but has been used off-label for nAMD treatment since 2005 [22]. The absence of routine pharmacovigilance and the general risks of compounding and off-label use of medications not intended for ocular use all add to the still partially unknown safety profile of bevacizumab for the treatment of retinal disorders, when compared with licensed anti-VEGF agents [23].

Ranibizumab has been licensed since 2006 for the treatment of nAMD [13], based on the efficacy demonstrated in the key trials, ANCHOR and MARINA, which were the first Phase III trials to show improvements in VA outcomes for all subtypes of CNV secondary to AMD [10, 17, 24]. Subsequent clinical trials, including BRAVO, CRUISE, RISE, and RIDE, demonstrated the efficacy of ranibizumab on a fixed, monthly dosing regimen across multiple indications [10, 11, 17, 24–30].

In some instances, the efficacy of both the 0.3 mg and 0.5 mg doses were compared when administered on a monthly regimen. In ANCHOR, VA outcomes at 24 months with monthly 0.3 mg ranibizumab versus monthly 0.5 mg ranibizumab were +8.1 and +10.7 Early Treatment Diabetic Retinopathy Study (ETDRS) letters respectively [11]. In MARINA, VA outcomes at 24 months with monthly 0.3 mg ranibizumab versus monthly 0.5 mg ranibizumab were +5.4 and +6.6 ETDRS letters respectively [17]. In BRAVO, which investigated the efficacy of ranibizumab in patients with macular edema following BRVO, the mean changes in best-corrected visual acuity (BCVA) from baseline to month 6 were 16.6 and 18.3 letters in the 0.3 mg and 0.5 mg groups respectively [31]. Similarly, in CRUISE, which investigated the efficacy of ranibizumab in patients with macular edema following CRVO, the mean changes in BCVA from baseline to month 6 were 12.7 and 14.9 ETDRS letters in the groups treated with 0.3 mg or 0.5 mg ranibizumab respectively [32]. In a pooled analysis of RISE and RIDE, 36-month VA outcomes in patients with visual impairment due to DME were +12.4 and +11.2 ETDRS letters with the 0.3 mg monthly dose and the 0.5 mg monthly dose respectively [29]. These studies highlight the comparable efficacy of the two doses when administered on a monthly regimen.

Following on from this, the European registration trial for ranibizumab in DME, RESTORE, focused on the 0.5 mg dose, but administered via a pro re nata (PRN) regimen, following three monthly loading doses [33]. The BCVA at the primary endpoint at 12 months was 6.1 ETDRS letters in the group randomized to ranibizumab from the beginning, and was 8.0 letters at 36 months (following the 2-year extension study) [34].

Intravitreal aflibercept (EYLEA^®^) was licensed for the treatment of nAMD in 2011 [13]. As well as inhibiting all isoforms of VEGF-A, aflibercept binds and inhibits VEGF-B and placental growth factor [35–37], and may also have other potential targets [38]. The VIEW trials demonstrated that in patients with nAMD, three initial monthly doses of intravitreal aflibercept 2.0 mg followed by dosing every 2 months delivered clinically equivalent outcomes to monthly ranibizumab (0.5 mg), but with five fewer injections in year 1 [39]. From weeks 52 through to 96, patients received their original drug assignment via a PRN-type regimen, in which therapy was administered as needed, with defined retreatment criteria and mandatory dosing at least every 12 weeks [40]. By week 96, the mean change in BCVA from baseline was a gain of 7.6 ETDRS letters in the groups previously assigned to intravitreal aflibercept dosed either monthly or every 2 months (bimonthly).

Intravitreal aflibercept is also licensed for the treatment of visual impairment due to mCNV, and due to macular edema secondary to DME or RVO. The VIBRANT, COPERNICUS, and GALILEO clinical trials all demonstrated the efficacy of intravitreal aflibercept in patients with macular edema following BRVO and CRVO, and showed that treatment with intravitreal aflibercept resulted in rapid vision gains of 12–13 letters after the first injection alone, with these gains maintained for at least 1 year [41–44]. Similar gains were also demonstrated in the MYRROR study in mCNV, where patients treated with intravitreal aflibercept were gaining an average of 13.5 letters by week 48 [45].

The VIVID and VISTA studies investigated the efficacy of intravitreal aflibercept in patients with DME. Not only were gains in vision maintained to week 100 (e.g., in VISTA, mean gains in BCVA from baseline to week 100 were 11.5 and 11.1 letters, with intravitreal aflibercept dosed monthly or bimonthly respectively [46]), but signs of diabetic retinopathy also regressed in patients treated with intravitreal aflibercept [46, 47].

A head-to-head comparison of anti-VEGF agents in the treatment of DME was conducted by the Diabetic Retinopathy Clinical Research Network [48, 49]. Using strict optical coherence tomography (OCT)- and vision-driven monthly retreatment criteria, the mean gains in VA from baseline to the primary endpoint at 12 months were 13.3, 11.2, and 9.7 with intravitreal aflibercept, ranibizumab, and bevacizumab respectively (*p* < 0.001 for intravitreal aflibercept versus bevacizumab, and *p* = 0.031 for intravitreal aflibercept versus ranibizumab) [49]. In particular, intravitreal aflibercept treatment was associated with numerically better outcomes in patients with a baseline VA of less than 69 ETDRS letters, with statistically significant results for intravitreal aflibercept (*p* < 0.001 for intravitreal aflibercept versus bevacizumab, and *p* = 0.003 for intravitreal aflibercept versus ranibizumab). This positive trend continued to 24 months, where VA outcomes in this subgroup of patients were 18.1, 16.1, and 13.3 letters, with intravitreal aflibercept, ranibizumab, and bevacizumab respectively (*p* = 0.02 for intravitreal aflibercept versus bevacizumab, and *p* = 0.18 for intravitreal aflibercept versus ranibizumab) [48].

Each anti-VEGF agent is likely to require a specifically optimized treatment regimen, but this must be balanced against the practicalities and costs of implementing each regimen in the clinic. Anti-VEGF agents have now been in ophthalmic use for the past 10 years [17], and the wealth of data from clinical and real-world studies support the clear, positive benefit–risk balance associated with their use for treatment of retinal disorders [50, 51].

### Anti-VEGF therapy in clinical practice

In each indication (excluding mCNV), the best outcomes have been shown in the clinical trials that employ fixed dosing [39]. Such a regimen is predictable and therefore straightforward for both the clinic and the patient. However, these regimens are usually associated with high clinic and patient burden (especially with monthly visits), and with risks of either over- or under-treatment if the fixed intervals between treatments are too short or too long [52]. For instance, the EXCITE study investigated the efficacy of ranibizumab 0.3 mg dosed on a monthly or quarterly regimen, following a loading dose of three consecutive monthly injections [52]. At month 12, the mean changes in BCVA were 8.3 and 4.9 ETDRS letters with the monthly and the quarterly doses respectively.

Numerous studies (especially with ranibizumab) have shown the challenges of bringing the efficacy demonstrated in clinical trials into the real world [53–57]. Given that anti-VEGF therapies for the treatment of DME were only approved in 2012, robust studies on the real-world effectiveness of anti-VEGF therapies for this indication have not yet been published. Nevertheless, translating the outcome achieved with fixed dosing in clinical trials will be difficult in clinical practice. Clinical practice has often been a compromise; fixed, monthly dosing is associated with huge burdens for both the patient and the clinic. Therefore, in real-world practice, PRN, treat-and-extend, and other regimens are adopted.

In PRN-type regimens, patients are only treated on disease reactivation, in an effort to reduce injection frequency and costs. Patients typically follow a monthly schedule of clinic visits, where the decision to re-treat at each visit is based on a set of prespecified criteria, as determined by the physician (e.g., VA and OCT/fluorescein angiography assessments) [58, 59]. Outcomes obtained with this regimen depend largely on the retreatment criteria employed by the treating physician and how closely these are adhered to. For example, Protocol T, which employed the use of strict retreatment criteria, saw favorable outcomes for ranibizumab and intravitreal aflibercept at the end of year 1 in patients with DME [49], and highlights the need to define, interpret, and adhere to retreatment criteria. RESTORE, however, which used predominantly VA-based retreatment criteria, resulted in substantially less VA gain at the end of year 1 [33]. Although cross-trial comparison is difficult due to differences in patient characteristics, it should be noted that even in a clinical trial setting some physicians will not adhere to the retreatment criteria, resulting in under-treatment of patients.

**Fig. 1:**
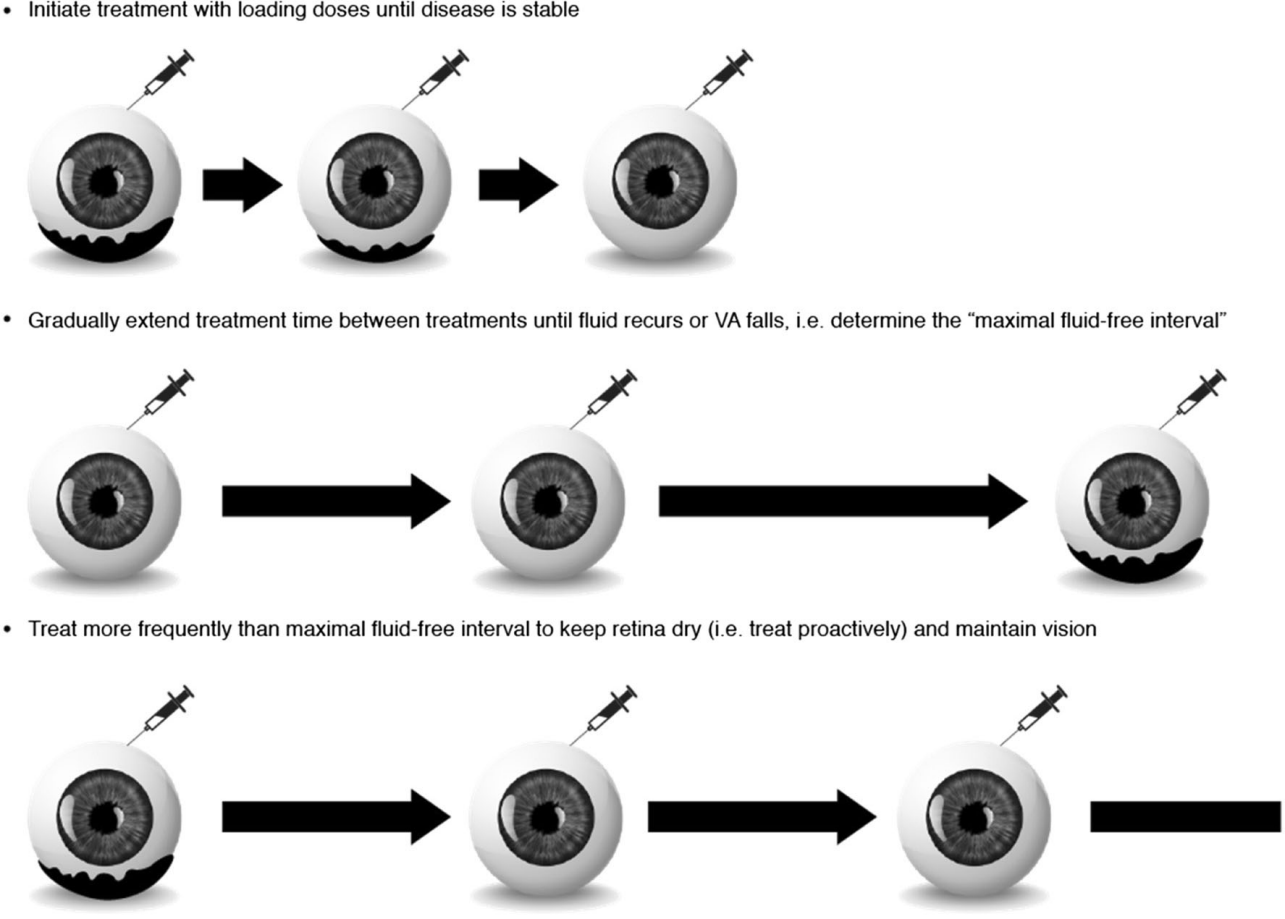
Illustrative example of how a treat-and-extend regimen might be implemented in the clinic

A treat-and-extend regimen is a more proactive and customized approach to treatment, with the goal of preventing disease recurrence. The drug is administered at every scheduled visit, regardless of visual or anatomic status on the day. However, the interval between each visit is either increased or decreased according to the anatomic and VA status, to determine the maximum time between injections without disease recurrence, i.e., the maximum recurrence-free interval (Fig. 1 gives an illustrative example of how a treat-and-extend regimen might be implemented in the clinic). The interval between treatments is then kept at a slightly shorter duration than that of the maximum recurrence-free interval to minimize the possibility of disease recurrence [13, 60] and maximize the length of time the patient receives protection from the drug. This approach reduces the frequency of clinic visits and removes the requirement for interim monitoring between injections, which helps to make the disease more manageable for the patient and physician while still ensuring that an appropriate number of injections are administered. This may also reduce the anxiety experienced by patients who fear disease recurrence, as well as any uncertainty regarding whether they will receive an injection at their forthcoming appointment [61]. The psychological and physical impact of any treatment regimen on patients is a factor that should always be considered.

The discrepancy in outcomes between clinical trials and the available real-world data is probably due to a number of factors.

Most physicians face organizational challenges related to clinic flow and follow-up frequency, usually due to resource limitations (including staff, VA assessors, and OCT machines). Reimbursement is also an issue, with some authorities imposing complicated approval procedures or limitations on patient characteristics.

Patient adherence to treatment regimens is another critical element. Although PRN regimens may help to reduce the number of treatments, many patients struggle to adhere to the necessary monthly monitoring schedule, which can result in wasted clinic resources. Patient adherence is itself affected by several factors, including age, psychological status, expectations of treatment, ease of travel, and cost (particularly where patients bear a large portion of the cost of treatment themselves).

## Methods

The Vision Academy Steering Committee met to discuss and define the focus of an ideal treatment regimen, and consider what would need to be done to help achieve this, if freed from the constraints of resource limitations or practical barriers. The practiced regimens and the associated advantages and disadvantages of each were initially reviewed. Data were examined from clinical trials and real-world studies, focusing on the licensed anti-VEGF agents used for the treatment of visual impairment secondary to nAMD, DME, and RVO. Literature searches were performed using the MEDLINE/PubMed database (cut-off date: March 2016) and restricted to English-language publications. To ensure a high level of evidence from the included data, studies with fewer than ten patients were excluded.

## Results: the principles of an ideal treatment regimen

Following a round-table discussion where the merits of each principle were considered, the Steering Committee consensus was that an ideal treatment regimen should encompass the following four principles:

1. Maximize and maintain VA benefits for all patients
2. Decide when to treat next, rather than whether to treat now
3. Titrate the treatment intervals to match patients’ needs
4. Treat at each monitoring visit.

These principles were developed with consideration of chronic retinal diseases that require regular therapy, as opposed to those diseases requiring less frequent treatment and less rigorous management (e.g., treatment of mCNV [27, 45]).

### Maximize and maintain VA benefits for all patients

Maximizing and maintaining VA for all patients should be a fundamental principle of any treatment regimen. The impact of improved and maximized VA on quality of life for patients is an important consideration, with improvements in BCVA shown to have a significant effect on a patient’s functional abilities [62]. A 5-letter increase in VA in the best-seeing eye has been shown to nearly double the likelihood of a patient being able to read a newspaper, and drive at night or under difficult conditions [62]. The significance of achieving driving vision for patients should not be underestimated; driving ability is often seen as an important measure of independence [63–65].

Clinical trials demonstrate that significant gains in VA are possible, and that these can be maintained in the long term. This is true across all key intravitreal aflibercept trials, including VIEW 1 and 2 [40, 66], COPERNICUS and GALILEO [42, 43], VIBRANT [67], and VIVID and VISTA [46]. In these studies, VA gains with intravitreal aflibercept were maintained over at least 1 year, and in some instances, for 4 years [66] (manuscript in development). Similar outcomes were also seen across the key ranibizumab trials, including ANCHOR and MARINA [11, 17], BRAVO and CRUISE [31, 32], and RISE and RIDE [29], which demonstrated improvements in and maintenance of gains in VA over 1–3 years. These results demonstrate that good visual outcomes are achievable, when fixed frequent dosing regimens are properly adhered to and treatment is administered as specified.

To date, however, these results have not typically been reflected in the clinic when PRN regimens are employed, with patients experiencing mean changes of between −0.8 and +3 letters following 12 months of ranibizumab treatment [53–57].

The UK Electronic Medical Record (EMR) Users Group analyzed patient records from 12,951 eyes of 11,135 nAMD patients across the UK, and showed that there was a mean gain of only 2 ETDRS letters over the first year of treatment with ranibizumab, and that these initial gains were not maintained beyond the first year [53]. In this study, the mean number of injections administered in the first year was 5.7, but this dropped to 3.7 in year 2.

**Fig. 2:**
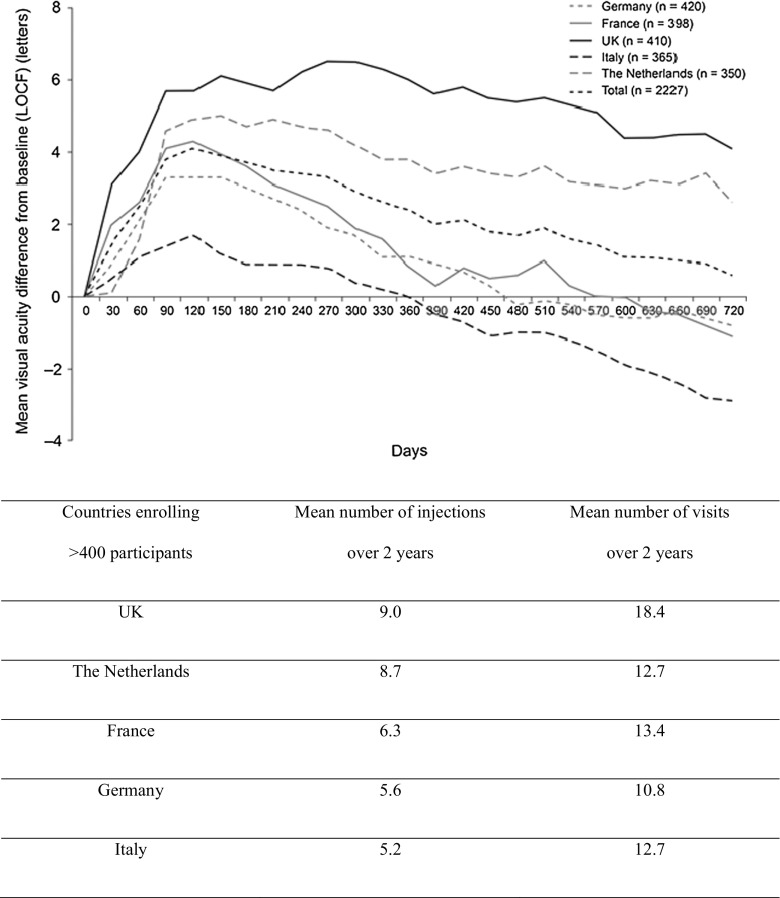
Outcomes from AURA: changes in VA from baseline per country over 2 years (only countries enrolling >400 participants have been displayed) [55]. *LOCF*, last observation carried forward

A more recent comprehensive review of 12-month outcomes as reported from real-world studies with ranibizumab for the treatment of nAMD also revealed that the weighted mean change in VA from baseline to month 12 was only +1.95 ETDRS letters [68]. In AURA (a retrospective, observational study conducted in Canada, France, Germany, Ireland, Italy, the Netherlands, the UK, and Venezuela), the medical records of 2,227 patients with nAMD treated with ranibizumab were evaluated [56]. The mean improvements in VA from baseline to years 1 and 2 were 2.4 and 0.6 letters respectively; the mean number of injections administered in the first and second year were 5.0 and 2.2 respectively (see Fig. 2 for outcomes stratified by country). It seems that unless a strict schedule of monthly monitoring visits and retreatment criteria can be adhered to [49], there is a risk that disease recurrence might not be detected sufficiently early for optimal treatment outcomes.

In DME too, suboptimal outcomes in VA will normally arise due to insufficient administration of therapy, leading to under-treatment. Comparing DME trials shows that when therapy is administered via a fixed dosing regimen [46], or a strict PRN regimen with clear retreatment criteria (Protocol T) [49], then gains in VA are maximized, with optimal outcomes for the patients. However, the results from RESTORE, REVEAL, and RETAIN [30, 33, 69], which have less strict PRN treatment criteria, show less favorable VA gains and fewer injections in year 1 compared with Protocol T. Similar outcomes are also observed through a comparison of trials with ranibizumab in nAMD, where PRN regimens are generally less effective than fixed dosing regimens [11, 17, 40, 70, 71].

A study by Oubraham et al. [72] highlighted the need for adequate treatment of all patients to achieve maximum gains in VA. This 12-month retrospective study investigated the efficacy of ranibizumab in nAMD patients, with therapy administered via either PRN (n=52) or treat-and-extend (n=38). Differences in VA gains between the two groups of patients were substantial, with those on the treat-and-extend regimen gaining a total of 10.8 ± 8.8 ETDRS letters vs 2.3 ± 17.4 ETDRS letters (*p* = 0.036) gained by patients treated according to the PRN regimen. This difference in outcomes was attributed to patients in the treat-and-extend group receiving significantly more injections than those in the PRN group (mean: 7.8 ± 1.3 vs 5.2 ± 1.9; *p* < 0.001), despite a similar number of follow-up visits (mean: 8.5 ± 1.1 vs 8.8 ± 1.5; *p* < 0.20). The group also tested the hypothesis that the difference in outcomes was driven by differences in the number of patients experiencing vision loss between the two groups; there were indeed significantly fewer patients with VA loss in the treat-and-extend group compared with the PRN group (2.4% vs 34.6%; *p* = 0.002).

Data from the AURA trial also provide a compelling example of the importance of maintaining a sufficient injection frequency as well as the importance of close monitoring. When results from the trial were stratified by country [56] (see Fig. 2), outcomes in Italy (where patients received the fewest injections) were worst, whereas outcomes in the UK (where patients had the highest number of injections) were far better, with greater improvements in VA. However, comparison of results from Canada with the UK reveal that while a similar number of injections were administered over 2 years (a mean of 9.9 vs 9.0, respectively), there was a greater difference in the number of visits (a mean of 13.6 vs 18.4, respectively). As a result, the mean change in VA at the end of year 2 was much lower in Canada (+1.6 ETDRS letters) than in the UK (+4.1 ETDRS letters), highlighting the importance of adequate monitoring in addition to a sufficient injection frequency. It should be noted that there was a difference in baseline VA between the two countries, with values of 47.2 ± 18.8 letters (Canada) and 55.0 ± 17.8 letters (UK).

Equally important to the concept of maximizing gains in VA is the early initiation of treatment, ideally within the first stages of disease onset. In the key intravitreal aflibercept trials in macular edema following RVO, immediate vision gains of 12–13 letters were seen after the first intravitreal aflibercept injection [67, 73, 74]. However, in COPERNICUS and GALILEO, the visual outcomes among patients initially randomized to receive 6 months of sham injections and switched to intravitreal aflibercept were inferior to those of patients who had been treated with intravitreal aflibercept from the outset [73–75]. For instance, in COPERNICUS, the mean changes in BCVA from baseline to week 100 in these two groups were +1.5 and +13.0 ETDRS letters respectively [75].

Similarly, a subgroup analysis of VIEW 1 and 2 highlighted the difficulty in trying to regain vision once it had been lost. A subgroup of patients in this trial were treated until stable and then only re-treated if vision deteriorated. Even if re-treated, this group never ‘caught up’ in terms of VA gains compared with those who were proactively treated [76, 77]. Certain subgroups of patients are also at a high risk of progressive visual loss if under-treated, e.g., approximately 50% of patients with newly diagnosed pigment epithelial detachment will experience significant visual loss (>3 lines) 1 year from diagnosis [78].

Also, Lim et al. [79] investigated the effects of a delay in treatment on VA outcomes. Results from 185 eyes with nAMD showed that a delay from initial signs suggestive of CNV to first injection was a significant predictor (*p* = 0.015) of poorer treatment outcomes (when controlling for age, sex, and baseline VA). In addition, a delay in treatment of 21 weeks or more compared with a delay of 7 weeks had an odds ratio of 2.63 (1.20, 5.68) for worsening vision after treatment.

It is clear from these studies that waiting for further disease progression before taking therapeutic action may be detrimental to the concept of maximizing VA gains, something that the early initiation of therapy is key to. Following this, maintenance of initial gains in VA must then be considered. Declines in VA are often seen in patients exiting these clinical trials [80], as demonstrated by the 5-year outcomes from CATT [70]. In this study, head-to-head comparisons between ranibizumab and bevacizumab were conducted when the two agents were administered via fixed and PRN regimens. After 2 years, patients were released from the clinical trial protocol, and were recalled for examination at 5 years. The mean change in VA at 5 years was −3 letters from baseline, and −11 letters from 2 years. This suggests that discrepancies in visual outcomes between clinical trials and real-world practice may be explained by changes in disease management. An example of this would be a change in the frequency of injections and/or monitoring.

The SEVEN-UP trial evaluated VA 7–8 years after initiation of ranibizumab therapy in 65 nAMD patients originally treated in the ANCHOR, MARINA, and HORIZON trials. Results showed a substantial decline in baseline VA, with one-third of patients experiencing a deterioration of 15 letters or more [80]. In this study, a mean total of 6.8 injections were administered over a mean 3.4-year period. One must also consider that patients who responded poorly to treatment would most likely have exited the initial studies, leaving only those who were experiencing better outcomes to continue the long-term study.

More promising results have been observed by the National Aflibercept UK Users Group, which documented visual and anatomic outcomes in 1,840 treatment-naive eyes of patients with nAMD who were treated with intravitreal aflibercept in accordance with the VIEW protocol. By the end of year 1, a median of eight injections had been administered over a median of eight visits per patient, resulting in a mean gain of 5.1 ETDRS letters [81].

A post-hoc analysis of VIEW [77] revealed that in the second year of treatment there was a subgroup of patients (approximately 20%) who suffered declines in vision when switching from fixed to PRN treatment. These results highlight the importance of considering the response to treatment in individual patients rather than the response observed in the whole patient population, as the latter may be driven by the best-responding patients. The evidence indicates that in order to maximize and maintain gains in VA across the patient population, regular treatment is necessary; deviations from this lead to suboptimal outcomes and a reduction in benefits for all patients, with the potential for significant vision loss in some individuals.

To summarize:

- Maximizing and maintaining gains in VA should be the aim of anti-VEGF treatment for all patients, not just those who respond well to therapy
- Early initiation of therapy and a sufficient frequency of injections are both essential for maximizing and maintaining gains in VA

### Decide when to treat next, rather than whether to treat now

In any disease, allowing for reactivation or unchecked progression typically leads to adverse consequences and progression of subclinical effects. These may be small or negligible at each event, but can accumulate and have significant long-term consequences, as demonstrated in patients with severe hemophilia A [82] and multiple sclerosis [83]. It is therefore good practice to avoid such episodes and stay ahead of the disease, adopting a proactive treatment approach, in which therapy is administered to minimize the risk of disease recurrence, rather than administered in response to it. In the management of retinal diseases, each clinic visit should therefore be seen as a treatment visit and an opportunity to decide, based on the current VA and anatomic status, when therapy should be administered next. These principles are at the heart of both fixed frequent and treat-and-extend treatment regimens.

The HORIZON trial investigated the efficacy of ranibizumab administered via an investigator-led PRN regimen in AMD patients who had previously exited (and completed) the ANCHOR and MARINA trials [84] (patients were also included from the FOCUS trial, which investigated the efficacy of ranibizumab combined with verteporfin PDT for the treatment of nAMD [85]). The switch from a strict monthly ranibizumab regimen to a more relaxed PRN regimen with less frequent follow-up not only resulted in worsening and progression of AMD in patients, but also a decline in the VA gains achieved on the monthly regimen [84].

In terms of improvements in VA and better outcomes for the patient, current evidence indicates that treat-and-extend regimens can also result in better visual outcomes compared with PRN regimens [72, 77, 86, 87]. A study by Hatz et al., in which 146 eyes from 134 treatment-naive patients with nAMD were switched from a PRN regimen to a treat-and-extend regimen, showed that greater improvements in vision were achieved following the switch in regimen [87]. After making the switch, not only did the mean BCVA (decimal) significantly improve from 0.49 at baseline to 0.55 and 0.56 at months 6 and 12 respectively (mean change in BCVA = 0.06, *p* < 0.001), but the mean central retinal thickness (CRT) also decreased from a baseline of 355 ± 112 μm to 330 ± 105 μm and 320 ± 103 μm at months 6 and 12 (*p* < 0.001) respectively. In addition, the mean number of visits declined during treat-and-extend. In the Oubraham et al. study previously discussed [72], nAMD patients on a treat-and-extend regimen demonstrated better outcomes in VA, with a significantly reduced risk of vision loss compared with their counterparts on the PRN regimen (2.6% vs 34.6%; *p* = 0.002), despite a similar number of visits.

These studies highlight the potential limitations of a PRN regimen; there may be a greater burden of appointments compared with a treat-and-extend regimen, but improvements in VA are likely to be suboptimal without frequent monitoring and strict retreatment criteria; achieving a successful PRN regimen therefore presents a significant challenge in clinical practice.

In a more recent study, patients with nAMD were treated with intravitreal aflibercept on a treat-and-extend regimen for 6 months, following prior treatment on a fixed bimonthly regimen for the first 12 months (with treatment initiated by three monthly loading doses) [88]. BCVA improved from 60.9 letters at baseline to 68.1 letters at month 12 (*p* < 0.001), and to 69.6 letters at month 18 (*p* < 0.001).

Additional evidence for treat-and-extend has been provided by ATLAS, a trial investigating the effects of intravitreal aflibercept administered via a treat-and-extend regimen in nAMD patients [89]. Over the course of 1 year, patients received a mean of eight injections, gaining between 10 and 11 ETDRS letters. By the end of the second year, an additional mean of 5.9 injections had been administered, and improvements in VA still stood at 8–11 letters.

The knowledge that an injection will be received at every visit and the predictable timing of the next injection could have a positive effect on patient experiences. Adherence to treatment regimens is crucial in clinical practice; better-informed patients not only experience reduced treatment burden but in turn have a positive impact on clinic operation. Knowing the timing of the next injection may also serve to maximize access to treatment in health systems where approval is required prior to the next injection, by allowing the physician more time to submit the necessary paperwork.

To summarize:

- The success of anti-VEGF treatment depends not only on the treatment of active disease, but also on the prevention of disease recurrence and/or worsening
- Planning the next anti-VEGF treatment helps to minimize the possibility of delays in treatment, allows time for treatment approval to be obtained if needed, and facilitates clinic management. Patients may also benefit from being able to anticipate and plan for their next injection in good time
- A proactive approach, such as treat-and-extend, allows physicians to stay ahead of the disease and, by minimizing the need for intervening visits, helps to ease the burden on clinics and patients

### Titrate the treatment intervals to match patients’ needs

**Fig. 3:**
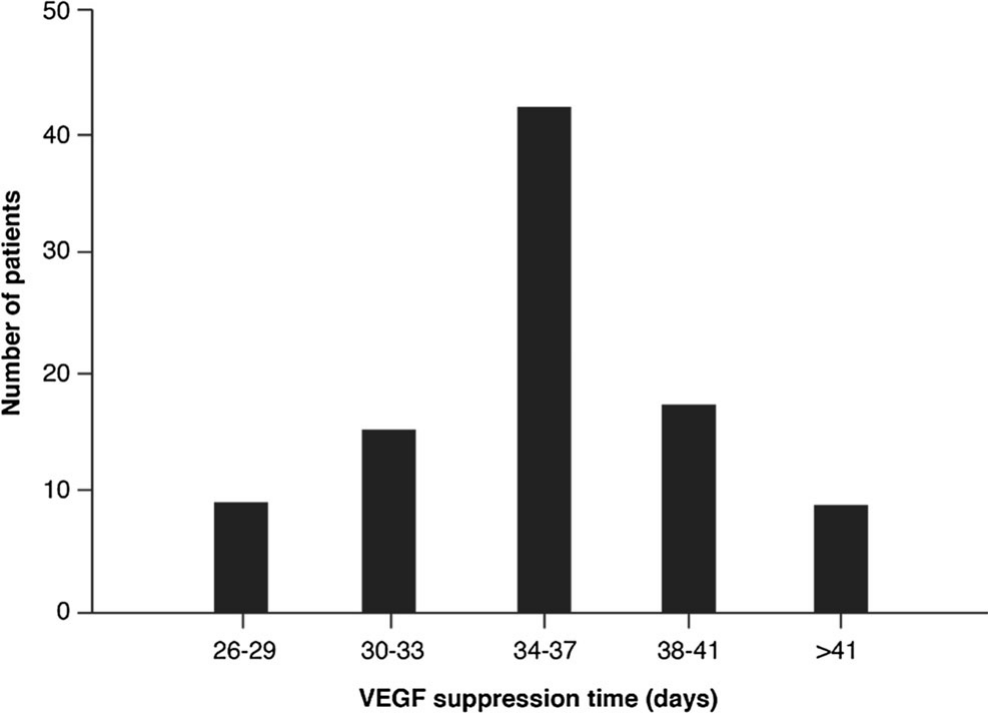
Distribution of individual VEGF suppression times of patients with nAMD treated with ranibizumab, as reported in Muether et al. [89]

The duration of VEGF-A suppression differs between patients and treatment. For example, intraocular measurements have revealed that VEGF-A suppression times in nAMD patients treated with ranibizumab can vary between patients from 26 to 69 days (Fig. 3) [90]. While different between patients, the suppression times were stable for each individual over a period of 3 years, with a mean suppression time of 36.4 days. This intra-individual stability has also been demonstrated in experiments investigating the suppression of VEGF by intravitreal aflibercept [91], which has been shown to suppress VEGF in the eyes of patients with nAMD for a mean of 71 days [91]. Although VEGF suppression times differ between patients, the consequences of this suppression are unclear. There are limited data available that link intraocular VEGF levels with outcomes; however, a recent study demonstrated that VEGF suppression time correlated with reductions in central retinal volume following treatment with either ranibizumab or aflibercept [92].

**Fig. 4:**
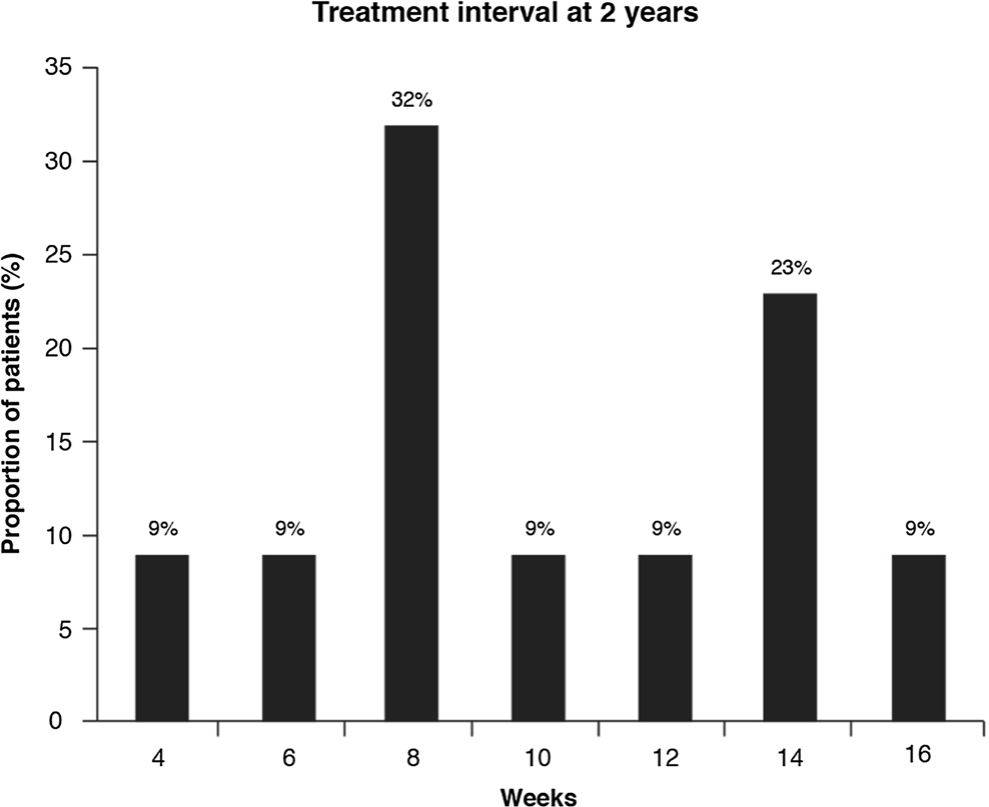
Distribution of treatment intervals in patients receiving intravitreal aflibercept in the ATLAS study [89]

Support for the biological difference between patients may also be found in clinical trial data. Post-hoc analyses of VIEW demonstrated that in the second year of treatment, approximately 48% of patients treated with intravitreal aflibercept required three or fewer injections between weeks 52 to 96 [77]. Similarly, in the second year of treatment in ATLAS, 82% of patients with nAMD had a treatment interval of 8 weeks or more, while 41% of patients had a treatment interval of 12 weeks or more [89] (see Fig. 4). The LUCAS trial also demonstrated differences in the mean number of treatments and intervals between nAMD patients treated with bevacizumab or ranibizumab administered via a treat-and-extend regimen, over the course of 2 years [93]. The percentage of patients achieving a treatment interval of 8 weeks or more was approximately 34% in the bevacizumab group and 47% in the ranibizumab group, while the percentage treated every 12 weeks was approximately 20% in the bevacizumab group and 33% in the ranibizumab group.

Mantel et al. [94] also investigated the predictability of the need for retreatment in nAMD patients. Treatment was initiated with three monthly loading doses of ranibizumab, after which patients were monitored on a weekly basis, with stepwise increases to every 2 weeks and then to monthly after each injection. Retreatment occurred on an as-needed basis. Results showed that the first interval between treatments after the loading phase was a good predictor of the following treatment intervals, and that there was high intra-individual predictability of retreatment need for patients with nAMD. This supports the notion of individualized treatment plans for patients, with a minimum number of injections and visits but maintained suppression of disease activity. Separate investigations support the idea that intra-individual stability of VEGF suppression is not exclusive to patients with nAMD, having also been observed in patients with DME [95].

Similarly, subgroup analysis of patients treated with intravitreal aflibercept in COPERNICUS and GALILEO revealed that after the initial 6-month period, some patients with macular edema following CRVO required fewer than three injections in the following 6 months to maintain vision [96]. Whether additional therapy would have led to improvements in VA is unknown.

A more individualized approach to treatment may encourage patients to adhere to treatment schedules by specifically tailoring the treatment regimen to their needs. Extension of intervals between treatments also sends a message that treatment is ‘working’, which may lift patients’ spirits and lead to renewed enthusiasm for treatment. This is in contrast with a PRN regimen, in which the marker for retreatment is anatomic recurrence or a decline in vision, which could be perceived as a ‘failure’ of treatment, thus negatively affecting patient psychology.

**Fig. 5:**
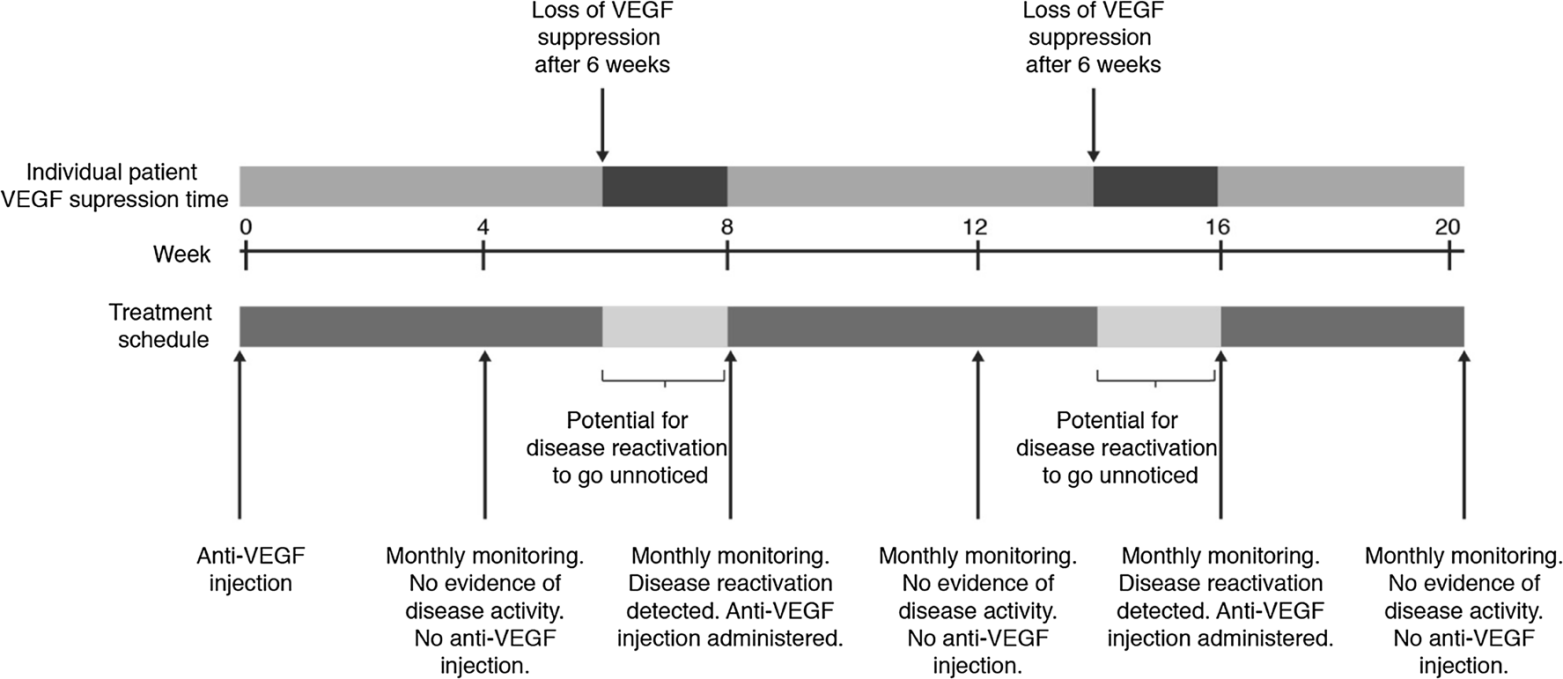
PRN treatment with monthly monitoring leaves patients at risk of disease resurgence

Physicians should be aware of the risks of PRN with monthly monitoring, which leaves the patient open to a potential risk of disease reactivation (see Fig. 5). To avoid this, an understanding of the intraocular durability of the various anti-VEGF agents is needed.

Studies in AMD, DME, and RVO have shown intravitreal aflibercept 2q8 to be equally as efficacious as intravitreal aflibercept 2q4 [40, 46, 97] and, in nAMD, also equivalent to ranibizumab 0.5q4 [40]. In a study by Pfau et al. [98], 13 patients with macular edema following CRVO requiring treatment with bevacizumab or ranibizumab at least every 6 weeks were switched to therapy with intravitreal aflibercept, administered via a treat-and-extend regimen. Twelve months after switching, the mean ETDRS letter score increased from 66.51 at baseline to 76.54 (*p* = 0.021), and the mean CRT decreased by 195.84 μm. The mean injection interval also increased by 0.51 months (*p* = 0.023) and the relapse-free interval by 3.02 weeks (*p* = 0.003).

The NEWTON trial [99] (which investigated the effect of switching from bevacizumab or ranibizumab to intravitreal aflibercept in patients with macular edema following CRVO) demonstrated that upon switching, the treatment interval could be extended from a mean 5–6 weeks to 8 weeks, with a 7.0-letter improvement in VA (*p* = 0.01) and a decrease in retinal thickness by 145 μm (*p* < 0.01). The average central macular edema–free interval also increased from 37 to 65 days (*p* < 0.0001).

This again links in with the idea of patient individuality and a difference in needs. By titrating the treatment interval to suit the patient’s needs and adjusting the treatment if necessary, physicians could achieve optimal outcomes for each patient through careful extension of the intervals between treatments, and aim to alleviate some of the burden on patients and clinics.

To summarize:

- The duration of VEGF suppression varies between patients and differs between anti-VEGF agents
- Anti-VEGF agents with greater duration of action might allow for longer extension of treatment intervals than for those with shorter durabilities
- Customization of the treatment interval to the individual patient removes the need for interim monitoring, while achieving optimal outcomes for the patient

### Treat at each monitoring visit

According to this concept, patients should be treated at every monitoring visit, meaning that no opportunity is missed to treat the disease. This simple change in practice has the potential to have a positive impact on the management of the disease; clinic flow would be improved by reducing the number of appointments per patient, and the possibility of unidentified disease recurrence would be minimized by the crucial elimination of any delay between assessment and treatment. Such delays (common in PRN regimens) often result in a change or worsening of disease status. Using each visit to collect the information required to enable treatment at the following visit should also help to ensure that treatment is delivered on time.

In a retrospective study by Oubraham et al. [72], the poor VA outcomes achieved by patients on a PRN regimen were attributed to a delay between identification and treatment of recurrences, which might potentially lead to irreversible foveal damage [100]. Such delays were avoided in the treat-and-extend arm of the study, in which the VA outcomes achieved were superior to those achieved by patients receiving treatment via the PRN regimen (particularly with respect to vision loss).

With consideration of the practicalities of managing multiple clinic appointments, scheduling one appointment for both treatment and monitoring should make it easier for patients to plan and manage travel to and from clinics, especially for patients who have long distances to cover or who need to be accompanied [61]. Furthermore, such a proactive approach, and the certainty that each clinic visit will involve an injection, may alleviate the stress experienced by some patients.

By using each clinic visit as an opportunity to monitor and treat, no chance is missed to stay ahead of the disease, and the physician will be able to make the most out of every interaction with the patient. This is important, as the literature shows that a delay in treatment or in treating recurrences of the disease can lead to suboptimal outcomes for the patient [72, 76, 77, 84].

While continued monitoring and treatment is part of the treat-and-extend strategy, no precise exit strategy has been defined for this approach as yet. The durability of anti-VEGF treatment has been demonstrated by long-term studies of up to 8 years [80], but further studies will be needed to determine whether treatment can be tapered and stopped completely following a treat-and-extend regimen.

To summarize:

- Monitoring and treating within the same appointment helps to eliminate the possibility of disease resurgence that can occur between separate monitoring and treatment appointments
- The number of appointments per patient will be reduced, helping to ease clinic flow and patient burden, and also reducing the stress experienced by the patient

## Discussion and summary

The ideal treatment regimen should be effective, proactive, individualized, and convenient. In summary, the following four principles are proposed as fundamental to an ideal treatment regimen for the treatment of chronic retinal disorders:

1. Maximize and maintain VA benefits for all patients
2. Decide when to treat next, rather than whether to treat now
3. Titrate the treatment intervals to match patients’ needs
4. Treat at each monitoring visit.

Together, the four principles point toward the implementation of a predictable, proactive and manageable treatment regimen, with consideration of individual patient needs and minimization of delays in treatment. If all four principles are implemented in practice, they are anticipated to lead to benefits for both patient and physician, with better organization of clinics, improved utilization of resources, and increased clinic capacity. Adopting a more personalized approach and reduced treatment burden may also lead to improvements in patient compliance.

It is hoped that implementation of a proactive regimen in intravitreal clinics will lead to further development and better understanding of appropriate treatment regimens, including treat-and-extend. There are few comparative studies that demonstrate the benefit of treat-and-extend, but the evidence base is growing, with further studies ongoing to fully characterize the impact of this approach.

While there is evidence for the benefits of treat-and-extend in nAMD (and RVO), DME is less clear, with treatment regimens in this disease area continuously evolving as more data come to light. The Protocol T data [49] highlight the effectiveness of aggressive PRN, in which the default position is to treat unless specific criteria are fulfilled. This is contrary to other PRN approaches such as in CATT [70], where treatment is withheld until certain criteria are met. It could thus be said that this remains a proactive treatment approach.

Little is also known about patients’ quality of life with treat-and-extend and PRN regimens, and discussions are needed on the obstacles that may be faced when integrating treat-and-extend regimens into existing clinical practice. There should also be further discussion of defining response to treatment, and when and how to stop treatment.

It is important to note that as an ‘ideal’ treatment regimen, costs of treatment (including country-specific financial drivers), have not been considered. These issues will need to be addressed on a local level, by taking the principles of an ideal regimen and holding discussions on a national basis on how best to implement them.

To date, it has proven challenging to translate the excellent outcomes seen in clinical studies into real-world practice. The Vision Academy, by recommending these four principles, anticipates that patient management can be improved, the burden on the clinic can be reduced, and outcomes for each patient can be optimized, with avoidable vision loss minimized.

## Appendix: Vision Academy Steering Committee

The Vision Academy Steering Committee is a group of ophthalmologists from across the globe, convened by Bayer to share best practice, knowledge, and lead the wider community in the drive toward optimized patient care. The Steering Committee of the Vision Academy comprises the following members:

Bora Eldem, Hacettepe University, Turkey

Alex Hunyor, University of Sydney, Australia

Antonia Joussen, Charité – University Medicine Berlin, Germany

Adrian Koh, Eye & Retina Surgeons, Camden Medical Centre, Singapore

Jean-François Korobelnik, University Hospital of Bordeaux, France

Paolo Lanzetta, University of Udine, Italy

Anat Loewenstein, Tel Aviv Sourasky Medical Center, Israel

Monica Lövestam-Adrian, Lund University Hospital, Sweden

Rafael Navarro, Institute of Ocular Microsurgery, Spain

Márcio Nehemy, Federal University of Minas Gerais, Brazil

Annabelle A. Okada, Kyorin University School of Medicine, Japan

Ian Pearce, Royal Liverpool University Hospital, United Kingdom

Francisco Rodríguez, Fundación Oftalmológica Nacional, Colombia

Sebastian Wolf, Inselspital, Switzerland

David Wong, University of Toronto, Canada
